# Fraction of Missing Information (*γ*) at Different Missing Data Fractions in the 2012 NAMCS Physician Workflow Mail Survey^*^

**DOI:** 10.4236/am.2016.710093

**Published:** 2016-06-15

**Authors:** Qiyuan Pan, Rong Wei

**Affiliations:** National Center for Health Statistics, Centers for Disease Control and Prevention, Hyattsville, MD, USA

**Keywords:** Multiple Imputation, Fraction of Missing Information (*γ*), Sufficient Number of Imputations, Missing Data, NAMCS

## Abstract

In his 1987 classic book on multiple imputation (MI), Rubin used the fraction of missing information, *γ*, to define the relative efficiency (RE) of MI as RE = (1 + *γ*/*m*)^−1/2^, where *m* is the number of imputations, leading to the conclusion that a small *m* (≤5) would be sufficient for MI. However, evidence has been accumulating that many more imputations are needed. Why would the apparently sufficient *m* deduced from the RE be actually too small? The answer may lie with *γ*. In this research, *γ* was determined at the fractions of missing data (*δ*) of 4%, 10%, 20%, and 29% using the 2012 Physician Workflow Mail Survey of the National Ambulatory Medical Care Survey (NAMCS). The *γ* values were strikingly small, ranging in the order of 10^−6^ to 0.01. As *δ* increased, *γ* usually increased but sometimes decreased. How the data were analysed had the dominating effects on *γ*, overshadowing the effect of *δ*. The results suggest that it is impossible to predict *γ* using *δ* and that it may not be appropriate to use the *γ*-based RE to determine sufficient *m*.

## 1. Introduction

Donald B. Rubin's 1987 book *Multiple Imputation for Nonresponse in Surveys* [1] is no doubt the most influential literature in the research field of multiple imputation (MI). It is in this book that Rubin described the parameter *γ*, which he termed as “the fraction of missing information”, and used it to define the relative efficiency (RE) of MI as [1]:

(1) $$RE={\left(1+\frac{\gamma }{m}\right)}^{\frac{1}{2}}.$$

Based on this RE, Rubin drew the following conclusion: If *γ* ≤ 0.2, even two repeated imputations appear to result in accurate levels, and three repeated imputations result in accurate levels even when *γ*=0.5 [1]. Anyone who wants to apply MI must first make a decision on what m should be considered sufficient for his or her program. Therefore, Rubin's conclusion of small m as being sufficient provided a possible answer to a question of fundamental importance and has been having a large impact on MI applications [2] [3]. As the key factor in RE, *γ* has been a very important concept in MI theory.

For a limited number of imputations in MI, *γ* is estimated by the following equation [1]:

(2) $$\gamma =\frac{r+2/(v+3)}{r+1}$$

where *r* is the relative increase in variance due to nonresponse and *v* is the degrees of freedom, defined by Equations (3) and (4) below, respectively [1]:

(3) $$v=(m-1){\left(1+\frac{1}{r}\right)}^{2}$$

(4) $$r=\frac{(1+\frac{1}{m})B}{U}$$

where *B* is the between-imputation variance and *U* is the within-imputation variance, defined by equations (5) and (6) below, respectively [1]:

(5) $$U=\frac{1}{m}\sum_{1}^{m}U_{i}$$

(6) $$B=\frac{1}{m-1}\sum_{1}^{m}{\left(Q_{i}-\bar{Q}\right)}^{2}$$

where the subscript *i* denotes the *i*th imputation in the MI and Q is the quantity of interest. The total variance (T) is:

(7) $$T=U+(1+m^{-1})B$$

As m approaches infinity, the following relationship can be deduced from Equations (2) to (7):

(8) $$\gamma_{m\to \infty }=\frac{B_{m\to \infty }}{B_{m\to \infty }+U_{m\to \infty }}=\frac{B_{m\to \infty }}{T_{m\to \infty }}$$

Equation (8) shows that *γ* is ultimately the fraction of *B* in *T*. *γ* is termed as “the fraction of missing information” probably because *B* would be otherwise missing from *T* unless MI is used [1].

In recent years, there is undeniable evidence that a much greater number of imputations, e.g. 40 or more, are needed in order to obtain reliable statistical inferences [4]-[11]. On the one hand, statistical software packages such as SPSS and SAS still uses *m* = 5 as the default value for the MI procedure, showing the persisting impact of Rubin's recommendation of small m as being sufficient. On the other hand, most researchers have realized that *m* ≤ 5 is too small and are now using 40 or more imputations in their MI applications [12]-[15].

Why would the apparently sufficient m as suggested by the *γ*-based RE be actually insufficient? The answer may lie with the characteristics of *γ*, for it is the only factor that defines the RE other than m itself. To date very little is known about the characteristics of *γ* other than what was described by Rubin in 1987 [1]. Even though many surveys are using MI, no published literatures can be found showing that *γ* values are determined using real survey data prior to the selection of sufficient *m*.

Fraction of missing information sounds similar to fraction of missing data (*δ*). How are *γ* and *δ* related? Rubin stated that *γ* would be equal to *δ* in the simple case of no covariates, and commonly less than *δ* when there are covariates [1]. However, the mathematical or empirical base for this statement on the *γ*-*δ* relationship is not given in Rubin's 1987 book or in any other published literatures.

Using the 2012 Physician Workflow Mail Survey (PWS12) of the National Ambulatory Medical Care Survey (NAMCS), the relationship between *γ* and *δ* is examined. The data presented in this paper add to our understanding of the characteristics of *γ* and may help explain why Rubin's *γ*-based conclusion on sufficient m may actually be too small. Results from the *m* = 80 MI trials on *γ*-*δ* relationship were presented at the 2015 JSM (the Joint Statistical Meetings) [16]. This paper represents more detailed findings, more thorough analyses and more comprehensive discussions on this topic at *m* = 99.

## 2. Methodology

Conducted by the National Center for Health Statistics (NCHS), the NAMCS Physician Workflow Mail Survey (PWS) was a nationally representative, 3-year (2011-2013) panel mail survey of office-based physicians, with each year being a complete survey cycle [17]. The data from the 2012 PWS, *i.e*. PWS12, were used in this research. PWS12 had 2,567 eligible, responding physicians in the sample. The MI trials had three experimental factors, *i.e*. *δ*,Imp_V, and Anal_V, referring to the fraction of missing data, the imputation variables and the data analysis treatments, respectively. Three variables representing the physician's practice size (SIZE), namely SIZE5, SIZE20 and SIZE100, were selected as the variables for imputation. SIZE100 is the practice size as represented by the number of physicians ranging from 1 to 100. SIZE5 and SIZE20 were derived from SIZE100. SIZE5 was derived by recoding the values of SIZE100 into 5 categories, and SIZE20 was derived by top-coding the values of SIZE100 greater than 20 into 20. These three variables were the values of Imp_V. They differed in their value ranges, distributions, and variances (Table 1).

Four levels of *δ*, *i.e*. 4%, 10%, 20%, and 29%, were used. PWS12 initially had 29% missing data due to item nonresponse for SIZE. After the missing values were replaced with non-missing values from the 2011 data for the same physician, the *δ* of PWS12 became 4%. The two other two *δ* values, 10% and 20%, were obtained by partially replacing, in a random manner, the missing values in 2012 with the non-missing values in the 2011 survey for the same physician. This method assumes that the value of SIZE would not change for the same physicians between 2011 and 2012. The method was officially used by NCHS in producing the public use data from PWS12. Therefore the *δ* values 4%, 10%, and 20% may be considered as the survey data instead of simulation data.

Hot deck imputation [18] was used. The statistics software package SAS 9.3 was used to carry out the imputation procedure. For each imputation variable at each *δ*, 1000 independent imputations were done. From this pool of 1000 imputations, samples were randomly drawn to form MI of various m values. For the purpose of this study, *m* = 99 was chosen, which was actually regarded as the *m* = 100 treatment. We used *m* = 99 instead of m = 100 because the SAS macro we developed for MI accepts one-digit and two-digit numbers only. To calculate the variance of *γ*, 30 random MI samples of *m* = 99 were drawn.

Anal_V had four values. They are CONTROL, REGION, PRIMEMP and DERIVED (Table 2). CONTROL is when no analytic variables were used in data analyses and is meant to be the control. REGION and PRI-MEMP are two real variables from PWS12. These two variables were used as the covariates in MI in the public-use data production for PWS12. DERIVED is a variable that was derived from and for SIZE5, SIZE20 and SIZE100 by regrouping the values of SIZE5, SIZE20 and SIZE100 into 4, 9, and 17 numerical categories, respectively, with the values at the group border line being randomly assigned to the two neighboring groups using SAS MOD function. The purpose of creating DERIVED is to have a variable that has high correlation with the imputation variables. REGION, PRIMEMP and DERIVED were used as the analytic variables in data analyses. Analyses were conducted with the un-weighted data.

## 3. Results and Discussions

### 3.1. The *γ* Values Were Strikingly Small

When Rubin deduced the sufficient m using the *γ*-based RE, the *γ* values listed in the table ranged from 0.1 to 0.9. When he concluded that *m* = 2 or 3 would be sufficient, he was assuming *γ* ≤ 0.5. He also mentioned that in the simplest case, *γ* could have the same value as *δ* [1]. Reading these descriptions of *γ,* people may expect that the magnitude of *γ* would be somewhat similar to *δ*. However, *δ* values obtained in this study were strikingly small, <0.01 in almost all the cases and could be as 10^−6^. Table 3 lists the mean *γ* values of different Anal_V and Imp_V combinations at *δ* = 10%. CONTOL for SIZE100 had the smallest *δ* mean, 0.000033, and DERIVED for SIZE100 had the largest *γ* mean, 0.009253 (Table 3).

### 3.2. Anal_V Had Dominant Effect on *γ*

The data used for this paper were from 1440 *m* = 99 MI trials resulted from a combination of 4 Anal_V treatments, 3 Imp_V variables, 4 *δ* levels, and 30 replicates. Analysis of variance (ANOVA) was performed, using PROC GLM of SAS software package, where GLM stands for the “general linear model”, to evaluate the overall effects of the three treatment factors, *i.e.* Anal_V, Imp_V and *δ*, and their interactions. Even though the *γ* values were small (Table 3), the variation of *δ* due to different experimental treatment was large, as indicated by the F values listed in Table 4. With an error DF (degrees of freedom) of 1392, the F value for reaching *α* =0.05 significant level is 3.00 for treatment DF = 2, and 1.60 for treatment DF = 18. However, even the smallest F value was as big as 461.1 in Table 4. All the effects of the three treatment factors and their interactions were highly significant (*α* < 0.0001). This suggests that, by a large m (*m* = 99) and sufficient number of replicates (30), the random errors were under a good control. More importantly, this shows that *γ* values can easily and greatly be affected by the three treatment factors (Table 4).

Judging by the magnitude of the F values, Anal_V was by far the most important factor affecting *γ*. It had a large F value of 23,158. The F value of the Anal_V × Imp_V interaction ranked the second, and that of Imp_V, the third. The primary purpose of this study was to investigate the *γ-δ* relationship. One may expect *δ* to have a major effect on *γ*. However the F value of *δ* was only one tenth that of Anal_V and one third that of Imp_V. Nevertheless an F value of 2223.9 for *δ* was still gigantic, indicating that *δ* can greatly affect *γ* (Table 4).

Anal_V's dominating effect on *γ* revealed by ANOVA can also be visualized by comparing the actual *γ* value changes attributed to *δ*, Anal_V and Imp_V. One way to do it is to compare the variation range of the *γ* means caused by different factors. Table 5 lists the maximum *γ* mean, the minimum *γ* mean and the range expressed as the percentage over the minimum for the three factors. The *γ* means at different *δ* varied a maximum of 105%, which is quite big. But this variation due to *δ* could easily be overshadowed by the effect of Anal_V, which caused as much as 13439% difference in *γ* means! The minimum *γ* mean and the maximum *γ* mean of *δ* were within the same order of magnitude, *i.e*. 0.001, whereas the minimum *γ* mean and the maximum *γ* mean of Anal_V were across three orders of magnitude, *i.e*. 0.00001, 0.0001 and 0.001 (Table 5). The effect of Imp_V as reflected by the variation in *γ* means was 135%, which was slightly greater than that of *δ* but much smaller than that of Anal_V (Table 5). Owing to the large impacts of other factors on *γ*, it is impossible to predict *γ* using *δ*.

### 3.3. Results of Regression Analysis

The linear model *γ* =*a* +*bδ* was applied to data of all Anal_V × Imp_V combinations. The values of the regression coefficient b and the results of the t test are presented in Table 6. The values of b were very small due to small *γ* values. It's t values, however, are very big: generally in two digits and highly significant at P < 0.0001 level. The largest t values was a remarkable number of 61.64, which occurred with the REGION for SIZE100. The large, positive t values indicate that *γ* would linearly increase with *δ*. There are exceptions, however. The t value for DERIVED with SIZE100 combination is negative (−4.22) and very significant (P < 0.0001), indicating a strong tendency for *γ* to decrease with the increase in *δ* in this particular case. The b value and the t value for DERIVED with SIZE20 were negative too (t = −1.81, p = 0.07) (Table 6).

### 3.4. Effects of Anal_V on *γ-δ* Relationship

**Figure 1:**
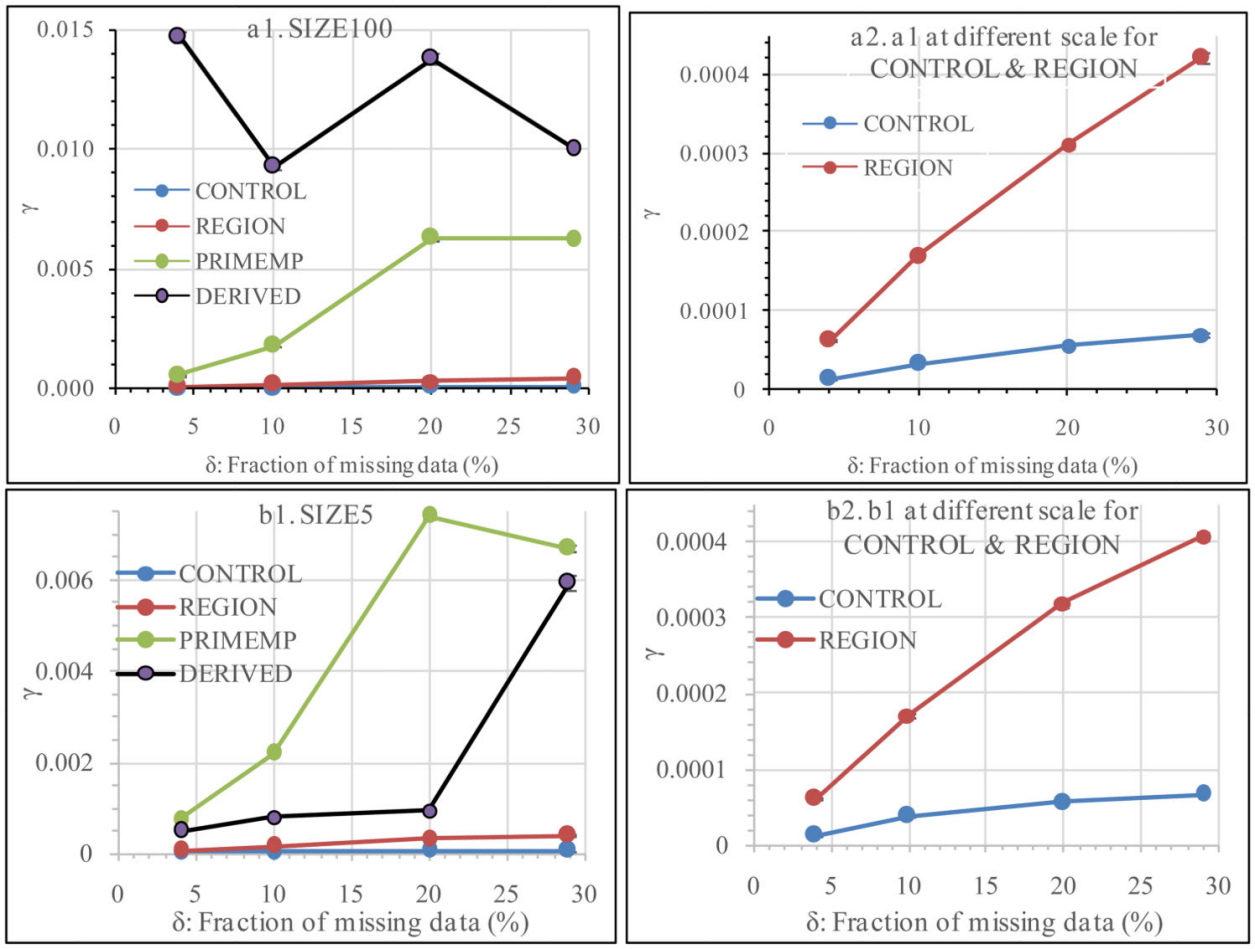
Effects of Anal_V on the *γ*-*δ* relationship. Since CONTROL and REGION had much smaller *γ*, they are graphed at different scale for better visualization in a2 and b2.

Figure 1 graphically shows how *γ* would change as *δ* increased from 4% to 29%. The four Anal_V treatments were presented in the same graph to make it easier for readers to visualize the main effect of Anal_V on *γ-δ* the relationship. CONTROL and REGION had much smaller *γ* values than PRIMEMP and DERIVED. They were graphed at a different scale in graphs a2 and b2 so that their *γ-δ* curves can be better visualized. The change patterns of *γ* with increased *δ* were drastically different among the four analytic treatments, indicating that how the data were analysed had major effect on the *γ-δ* relationship (Figure 1(a1) and Figure 1(b1)). For CONTROL and REGION, *γ* increased linearly with the increase of the *δ*(Figure 1(a2) and Figure 1(b2)). For PRIMEMP and DERIVED, however, the *γ-δ* curve might increase, *zigzag*, remain flat, or decrease in a highly significant (*α* < 0.01) manner with the increase of *δ* (Figure 1(a1) and Figure 1(b1)). The effects of PRIMEMP and DERIVED on the *γ-δ* relationship were affected by Imp_V. For example, as *δ* increased from 4% to 29%, the line of DERIVED zigzagged in SIZE100 but never went downward in SIZE5 (Figure 1(a1) and Figure 1(b1)).

**Figure 2:**
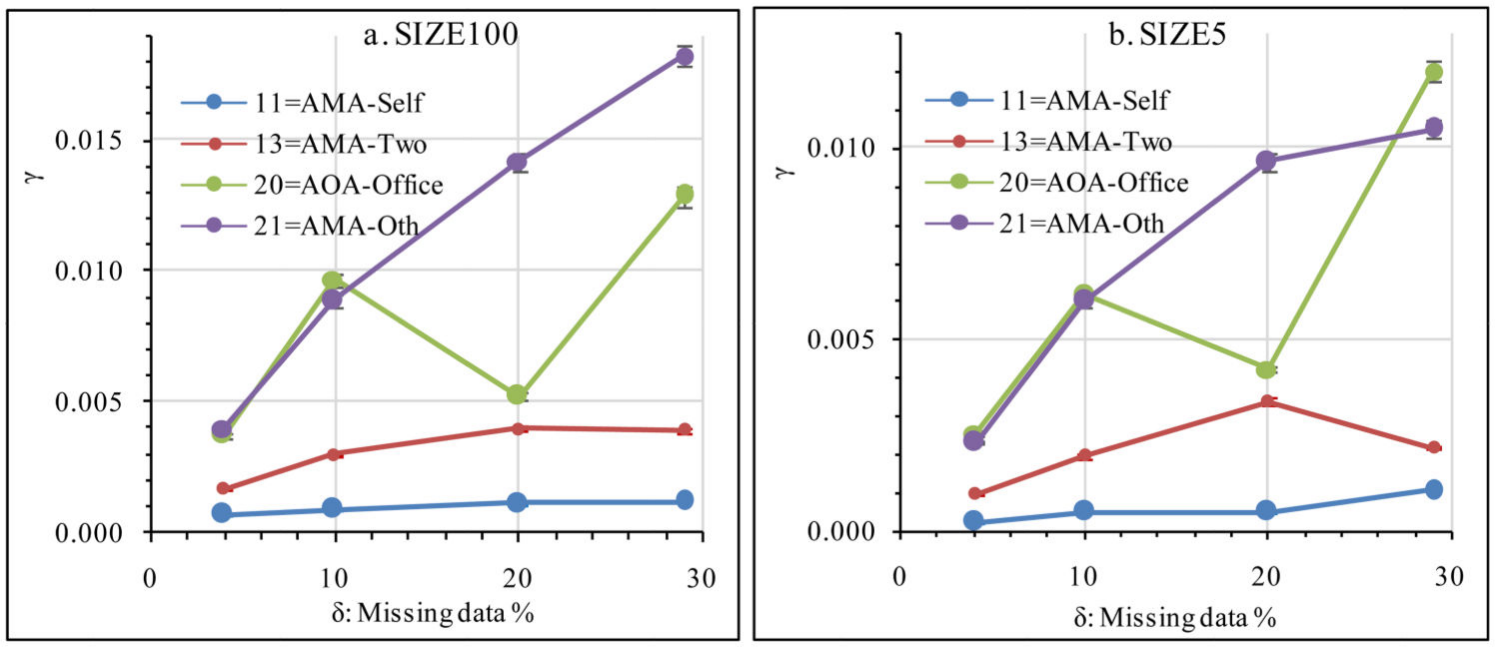
Effect of different categories of PRIMEMP on the *γ*-*δ* relationship. Data of the first four categories of PRIMEMP (Table 2) are presented.

*γ* values were also calculated for different values of an Anal_V treatment, for instance, Northeast vs. West in REGION. Would different levels or categories of the same Anal_V treatment have different *γ* values and *γ-δ* relationships? Data of the first four categories in the value list of PRIMEMP (see Table 2) are presented for SIZE100 and SIZE5 in Figure 2. The four PRIMEMP categories had different *γ* values and *γ-δ* relationships, as visualized by the line graphs of Figure 2. When *δ* increased, *γ* usually increased but could decrease or remain unchanged (Figure 2). For the same PRIMEMP category, the line heights (indicating *γ* values) and shapes (indicating the *γ-δ* relationship) were similar between SIZE100 (Figure 2(a)) and SIZE5 (Figure 2(b)).

### 3.5. Effects of Imp_V on *γ-δ* Relationship

**Figure 3:**
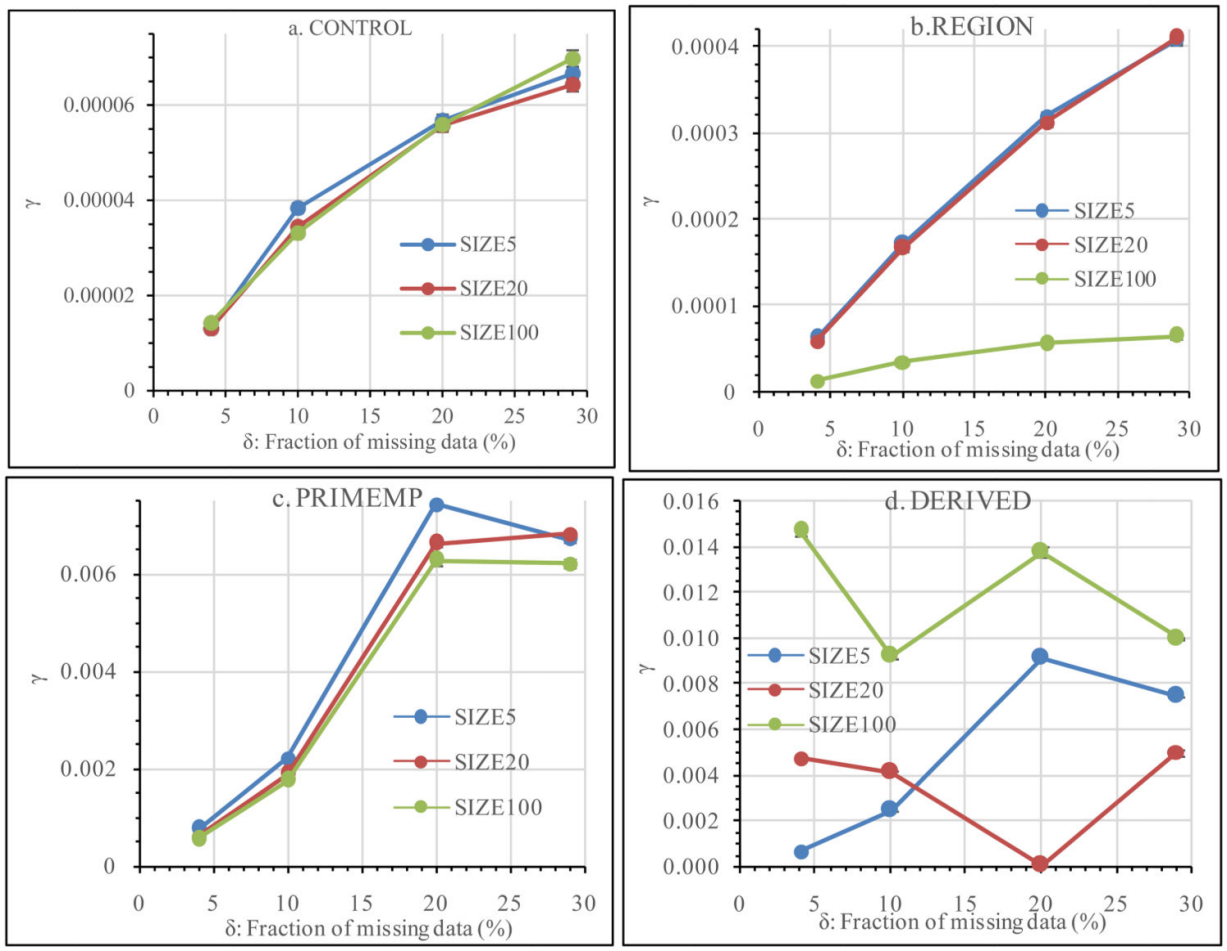
Effects of Imp_V on the *γ-δ* relationship.

Figure 3 shows the effect of Imp_V on the *γ-δ* relationship. The difference among SIZE5, SIZE20 and SIZE100 had different patterns with different Anal_V treatments. When Anal_V= CONTROL, the *γ-δ* lines were similar among SIZE5, SIZE20 and SIZE100 (Figure 3(a)). When Anal_V= REGION, the *γ-δ* lines were almost identical between SIZE5 and SIZE20, but *γ* values were lower and the slope of the *γ-δ* line was smaller for SIZE100 (Figure 3(b)). When Anal_V= PRIMEMP, the general trends of the *γ-δ* lines were similar among SIZE5, SIZE20 and SIZE100 (Figure 3(c)). Large differences in the *γ-δ* relationship existed among SIZE5, SIZE20 and SIZE100 when Anal_V = DERIVED (Figure 3(d)). In summary, Imp_V might or might not have significant impact on the *γ-δ* relationship depending on how the data would be analysed.

## 4. Additional Discussions

### 4.1. Why Were *γ* Values So Small?

From the Equations (2) to (7), we see that when *m* → ∞, *γ* becomes *B/T*, where *T* = *B* +*U*, as indicated by Equation (8). The *m* value chosen for the MI trials in this study, *m* = 99, is very large comparing to what Rubin recommended [1]. *m* = 99 could be regarded as an approximation of *m* → ∞. PWS12 had a sample size of *n* = 2567. The “quantity of interest”, *i.e*. *Q* for Equation (6), is the sample mean. From Equations (5) and (6), we can see that *B* is the variance of the sample means, and *U* is the mean of the sample variances. Based on the classic statistics, the sample variance *s*^2^ and the variance of the sample means $s_{\bar{x}}^{2}$ has the following relationship [19]:

$$s_{\bar{x}}^{2}=s^{2}/n$$

If all 2567 values were randomly drawn during the MI process, then $s_{\bar{x}}^{2}$ can be approximated by *B*, and *s*^2^ can be approximated by *U*, and we will have the following relationships:

$$B=\frac{U}{2567}$$

and

$$\gamma =\frac{B}{T}=\frac{U}{2567T}$$

Since *U* ≤ *T*, we will have *γ* ≤ 1/2567 = 0.00039. The assumption that all values are regenerated by the imputation process would be possible only if *δ* = 100%. The largest *δ* in this study was 29%, meaning that 71% of the values would be identical among the 99 samples generated by MI. *B* and *γ* would be smaller than if the all the values were generated by MI. This may help explain why the *γ* values were so small.

### 4.2. The Implications of Small *γ*

Rubin's conclusion of *m* ≤ 5 as being sufficient assumes *γ* ≤ 0.5. For a long time, researchers who adopted a small m in their MI programs may have had a concern that their *γ* might be greater than 0.5 so that Rubin's requirement for small m as being sufficient was violated. The result of this study suggest that *γ* may never be greater than 0.5. Now that we no longer need to worry about *γ* being too large, should we use just a few imputations in our MI without any other concerns? The answer would be a “Yes” only if we accept that Rubin's *γ*-based RE can indeed be legitimately used for determining the sufficient m for MI under any circumstances.

Should the *γ*'s being too small be a concern? The *γ* for PWS12 as determined in this study was <0.01. With such small RE, Rubin's *γ*-based RE will assume a value of 1 even at *m* = 1, meaning that a single imputation is sufficient. With such a small *γ*, we may conclude that MI is meaningless. Or, alternatively, we may conclude that the *γ*-based RE is inappropriate for determining the sufficient m for MI. Nowadays MI is a popular approach in dealing with missing data [2]. To deny MI entirely just because of the smallness of *γ* does not seem to be reasonable, for there may be other ways to define RE of MI and there may be other ways to determine the sufficient m for MI [5].

### 4.3. Are *γ* and *δ* Comparable?

Rubin made the following statement in his 1987 book [1]: “The quantity of *γ*_0_ is equal to the expected fraction of observations missing in the simple case of scalar *Y_i_* with no covariates, and commonly is less than the fraction of observations missing when there are covariates that predict *Y_i_*,” where *γ*_0_is the same as the *γ* in Equation (1), Y is the imputed variable and Y_i_ is the value of Y for the *i*th unit. This statement about the *γ-δ* relationship might lead people to have a feeling that *γ* and *δ* are linked, comparable, and similar in magnitude. In this study, we see that *γ* was one, two, or sometimes three orders of magnitude smaller than *δ*. This enormous difference between *γ* and *δ* cannot be possibly explained by the existence or absence of covariates as suggested by Rubin [1].

As indicated by Equation (8), *γ* is essentially a ratio of variances, whereas *δ* is simply a ratio of sampling unit counts. After the survey is completed, *δ* is a fixed value, whereas *γ* is still undecided prior to the MI and even after the MI is completed. As a ratio of variances, *γ* may be regarded as the second order of statistics which involves a squaring of the first order of statistics such as the sample mean, the standard deviation, and the sample size. On the other hand, as a ratio of sampling unit counts, *δ* belong to the first order of statistics. The unit of *γ* would be at the same order as *δ* if we take the square root of *γ*. For CONTROL, when *δ* = 4%, 10%, 20%, and 29%, the corresponding *γ* means were 0.000013, 0.000035, 0.000056, and 0.000067, respectively, and the corresponding √*γ* values were 0.003673, 0.005944, 0.007493, and 0.008181, respectively. Therefore, even if we take the square root of *γ*, the parameter √*γ* could still be very different from *δ*. The results of this study suggest that *γ* and *δ* have totally different meaning and units and are not comparable.

As we see from Equations (2) to (6), *δ* is not a factor in defining *γ*. Data from this study suggest that even though *δ* has significant effect on *γ*, it is far from being a dominant factor. It may almost be impossible to mathematically relate *δ* to *γ*. The conditions for *γ* = *δ* has not been mathematically visualized in the published literature as well as in this study. Given the fact that the effect of *δ* on *γ* can be easily overshadowed by other factors, it is impossible to predict *γ* using *δ* even if a linear relationship may exist between *γ* and *δ* under certain circumstances.

## 5. Conclusive Summary

Using the real survey data from PWS12, MI was performed at *m* =99 and *δ* = 4%, 10%, 20%, and 29% for Imp_V= SIZE5, SIZE20, and SIZE100. The *γ* valued were determined for Anal_V = CONTROL, REGION, PRIMEMP and DERIVED. The following conclusions may be drawn from the results and discussions of this study:

1. *γ* and *δ* have different meaning and units and are not comparable. *γ* is essentially a ratio of variances, whereas *δ* is a ratio of the counts of sampling units. *δ* is a fixed value once the survey is complete, whereas *γ* is not known prior to and after the MI.
2. Anal_V had the dominating effect on *γ* as compared to other two factors tested, *i.e.* Imp_V and *δ*. The variation of *γ* due to Anal_V was 100 times greater than that due to *δ*, judging by the range of *γ* means. The effect of Imp_V was much smaller than that of Anal_V but greater than that of *δ*.
3. The linear increase of *γ* with increased *δ* was observed. The decrease of *γ* with increased *δ* was also observed. Even though the linear regression coefficients were highly significant (P < 0.0001) in the majority of cases, it may not be possible to predict *γ* using *δ* because the effect of *δ* can easily be overshadowed by the effect of Anal_V and Imp_V.
4. Rubin stated that *γ* would be equal to the expected *δ* in the simple case of no covariates, and commonly less than *δ* when there are covariates [1]. The magnitude of *γ* in this study varied from 0.01 to 0.000001 while *δ* varied from 4% to 29%. The enormous difference between *γ* and *δ* cannot possibly be explained by the presence or absence of covariates. The supposition that *γ* = E[*δ*] does not seem to be untenable.
5. The magnitude of *γ* obtained from this study was very small (<0.01). On the one hand, one may not need to worry about *γ* being too large (>0.5) when applying Rubin small-m recommendations. On the other hand, the smallness of *γ* may challenge the rationality to use the *γ*-based RE for determining the sufficient m. For *γ* < 0.01, a single imputation would be sufficient and MI would become meaningless if we use the *γ*-based RE to determine the sufficient *m*.

## Figures and Tables

**Table 1:** Characteristics of the imputation variables (Imp_V).

Imp_V	Description	Mean	Value range	Variance
SIZE100	Practice size as represented by the number of physicians.	11.41	1 - 100	483.02
SIZE5	Practice size recoded from SIZE100: 1 = Solo practice; 2 = Twophysicians; 3 = 3 to 5 physicians; 4 = 6 - 10 physicians;5 = 11+ physicians.	3.06	1 - 5	1.97
SIZE20	Practice size recoded from SIZE100: 1 - 19 = The actual number of physicians; 20 = 20+ physicians.	6.47	1 - 20	38.26

**Table 2:** Description of the analytic treatments (Anal_V).

Anal V	Description	Value range
CONTROL	No analytic variable	Not applicable
REGION	Region of the physician interview office	1 = Northeast, 2 = Mid West, 3 = South, 4 = West
PRIMEMP	Primary present employment of the physician	11 = AMA-Self-emp, solo prac; 13 = AMA-Two phy. prac; 20 = AOA-Office prac. solo; 21 = AMA-Oth pat care/AOA-Off prac. partnp; 22 = AOA-Office prac group; 23 = AOA-Offcpracofc employee; 30 = AMA-Grp prac/AOA-Off prac HMO staff; 31 = AOA-Office prac. walk-in clinic; 35 = AMA-HMO; 40 = AMA-Medical school; 64 = AMA-County/Cty/State Govt Other; 97 = AOA-other office or clinic practice; 110 = AMA-No classification; 200 = Sampled CHC
DERIVED	Derived categories for SIZE5, SIZE20, and SIZE100	Regrouping the values with random errors added to each group. 1 to 4 for SIZE5, 1 to 9 for SIZE20, 1 to 17 for SIZE 100

**Table 3:** Mean *γ* values for different Anal_V and Imp_V combinations at *δ* = 10% and *m* = 99.

Anal_V	Imp_V		

	SIZE5	SIZE20	SIZE100
CONTROL	0.000038	0.000034	0.000033
REGION	0.000171	0.000166	0.000169
PRIMEMP	0.002212	0.001876	0.001756
DERIVED	0.000805	0.004115	0.009253

**Table 4:** Results of ANOVA, showing the effects of *δ*, Anal_V, Imp_V, and their interactions on *γ*.

Source of variation	DF	Variance	F value	P > F
Model	47	0.00042800	3387.0	0.0001
*δ*	3	0.00028103	2223.9	0.0001
Anal_V	3	0.00292646	23158.3	0.0001
Imp_V	2	0.00077419	6126.6	0.0001
*δ* × Anal_V	9	0.00024690	1953.8	0.0001
*δ* × Imp_V	6	0.00005859	463.7	0.0001
Anal_V × Imp_V	6	0.00088711	7020.1	0.0001
*δ* × Anal_V × Imp_V	18	0.00005827	461.1	0.0001
Error	1392	0.00000013		

**Table 5:** Ranges of *γ* means for *δ*, Anal_V and Imp_V.

Item	*δ*	AnalV	Imp_V
Minimum *γ* mean (min)	0.0017	0.000043	0.0017
Maximum *γ* mean (max)	0.0035	0.005822	0.0040
Difference% = 100 × (max – min)/min	105	13439	135
Minimum *γ* mean (min)	0.0017	0.000043	0.0017

**Table 6:** The regression coefficient (b) and the corresponding t value for the linear model *γ* = *a* + *bδ* for different Anal_V × Imp_V combinations.

Anal V	Imp V	b (Regression coefficient)	t value for *H*_0_: *b* > 0	P for t
	SIZE5	0.00000206	26.88	<0.0001
CONTROL	SIZE20	0.00000202	29.58	<0.0001
	SIZE100	0.00000221	31.98	<0.0001
	SIZE5	0.00001383	53.64	<0.0001
REGION	SIZE20	0.00001403	59.82	<0.0001
	SIZE100	0.00001426	61.64	<0.0001
	SIZE5	0.00027159	23.19	<0.0001
PRIMEMP	SIZE20	0.00027602	31.38	<0.0001
	SIZE100	0.00025355	26.92	<0.0001
